# Annotations

**Published:** 1905-08-19

**Authors:** 


### ANNOTATIONS.

### Hygiene for the Army.

Sir H. Kimber raised an important question in Parliament last week. He asked whether the recommendations of the Advisory Medical Board, referring to the sterilisation of fluids used for drinking purposes in the Army during manœuvres, foreign and active service, had been carried out. He explained that the recommendations involved the provision of sterilising apparatus and the appointment of a trained staff in charge. Mr. Arnold-Forster denied that the Advisory Board made such sweeping recommendations; but he explained that trials and investigations in respect to methods and apparatus were proceeding, and since the question was raised in Parliament Mr. Arnold-Forster has given practical evidence that he is interested in the subject by inspecting various devices for sterilising water. It is consistent with British methods that five years should have elapsed since urgent recommendations for reform were made, and that the experiences of South Africa should have made so little apparent impression. In the meanwhile the Japanese with their usual perspicuity have attached the best brains and knowledge to their armies, and through the practical application of scientific methods, have largely discounted the perils of the campaign. And this achievement was probably even more difficult in Manchuria than it would have been in South Africa, where it is a fact that commanding officers were known to disregard the counsels and opinions of their medical comrades. Sir H. Kimber has brought the matter to public attention, and Mr. Arnold-Forster is evidently alive to its importance. It is now the duty of the public in the interests of the nation and its soldiers to insist that the labours of the Commission on Dysentery and Enteric Fever appointed in 1900 shall not be without fruit. Commissions are expensive, and too often they have no other result than serving as a sop to Cerberus.

### A French Consumptive Sanatorium.

Near Lille, in the heart of the "Black Country" of France, there has lately been established a sanatorium for tuberculosis which differs in several repects from any established in this country. As is well known, coal-miners suffer much from lung troubles, for, though the better ventilation of mines has rendered the life of a miner much less unhealthy than it was formerly, consumption is still one of the curses of the work. The cost of the sanatorium—£8,000—has been borne by the employers of labour in the neighbourhood of Lille. The sanatorium is really a village, and comprises a building for unmarried men, another for unmarried women, and 24 "villas," which form 48 houses for families. In England the idea is always to separate the consumptive from his family, for their protection as well as for his own profit. That this is ideally the best thing may be admitted; but, on the other hand, the idea of prolonged separation undoubtedly hinders many patients from entering a sanatorium until it is too late for the treatment to be of permanent benefit to them. A man works on until he can do no more, because he feels that his going away, even though of ultimate benefit to himself, will leave his family destitute, and when at last he accepts sanatorium treatment he is often too ill to get more than temporary good from it. Therefore there is common sense in the French notion of removing the whole family to healthy surroundings. Moreover, as the family of a consumptive are often by no means vigorous the change may be useful in strengthening them against the possibility of tuberculous infection. The rules of the colony demand that each patient shall take the rest and fresh-air cure for six hours a day, but the time may be divided at will so that nothing more than a frequent lounge is consciously demanded of him. In fact, this French village for consumptives seems intended to produce the maximum of benefit with the minimum of restraint. Whether the institution proves to be a practical success or otherwise time alone will show, but the spirit in which the rules and arrangements have been made is entirely commendable, and well worthy of emulation on this side of the channel.

### Voluntary versus Rate-supported Ambulances.

Recent discussions regarding the provision of an ambulance service for London and other large cities, render particularly valuable the paper read at the last meeting of the British Medical Association by Colonel H. J. Barnes, R.A.M.C. Colonel Barnes is the general secretary of the St. Andrew's Ambulance Association, and he argues in favour of an ambulance waggon service founded and maintained by voluntary effort on the part of the community in preference to a rate-supported organisation. He claims for the former an elasticity, flexibility, and freedom from red-tape which it is impossible to obtain from an official scheme. In support of his position he quotes the experience of the St. Andrew's Association in meeting the wants of the Scottish towns, and more particularly those of Glasgow. Since 1882, when the service was first commenced, the Scottish organisation has steadily increased, until in 1904 there were 18 towns supplied with 23 horse-drawn ambulances, and 6,287 cases were carried during the year without a single instance of complaint. The Glasgow service, covering an area of 17,605 acres and including a population of 925,000 persons, is supplied from a single stable, and five waggons are found sufficient for the work. With each ambulance, in addition to the driver, there travels a trained attendant competent to render first aid in the absence of a medical practitioner. No charge is made for the use of the waggons in cases of accident or of sudden illness occurring in the streets or other public places, and only a nominal sum is charged for the removal of cases of non-infectious illness from private houses to hospitals. Communication by telephone is effected without any special arrangements, and it is here, perhaps, that the weak point of the system exists. A "call office" may readily be found during the day time, but at night this is not the case, and even though police stations and cab offices may be available, a more prompt and accessible means of communication must often be desired. Altogether the Scottish plan seems to have great practical merits, to which reformers in other cities might wisely direct their attention.

